# Introducing RAPTOR: RevMan Parsing Tool for Reviewers

**DOI:** 10.1186/s13643-019-1070-0

**Published:** 2019-06-26

**Authors:** Lena Schmidt, Farhad Shokraneh, Kirsten Steinhausen, Clive E. Adams

**Affiliations:** 1 Fakultät Gesundheit, Sicherheit, Gesellschaft, Hochschule Furtwangen University, Robert-Gerwig-Platz 1, 78120 Furtwangen, Germany; 2 University of Nottingham, Nottingham, UK; 3 Institute of Mental Health, University of Nottingham Innovation Park, Jubilee Campus, Triumph Road, Nottingham, NG7 2TU UK

**Keywords:** RevMan, Automation, Systematic reviews, Data extraction, Document classification, Automatic document classification, Review Manager, Natural language processing, NLP, XML

## Abstract

**Background:**

Much effort is made to ensure Cochrane reviews are based on reliably extracted data. There is a commitment to wide access to these data—for novel processing and/or reuse—but delivering this access is problematic.

**Aim:**

To describe a proof-of-concept programme to extract, curate and structure data from Cochrane reviews.

**Methods:**

One student of Applied Sciences (16 weeks full time), access to pre-publication review files and use of ‘Eclipse’ to create an open-access tool (RAPTOR) using the programming language Java.

**Results:**

The final software batch processes hundreds of reviews in seconds, extracting all study data and automatically tidying and unifying presentation of data for return into the source review, reuse, or export for novel analyses.

**Conclusions:**

This software, despite being limited, illustrates how the efforts of reviewers meticulously extracting study data can be improved, disseminated and reused with little additional effort.

## Background

The Cochrane Library contains the largest repository of maintained systematic reviews [1]. It is created by the efforts of thousands of people, still mostly volunteers, carefully extracting data from studies (largely randomised trials), entering these qualitative and quantitative data into an open-access software package (RevMan [2]). RevMan undertakes analyses whilst marking up all text for full publication on the online Cochrane Library. RevMan data entry is laborious but Cochrane methods endeavour to maximise the reliability of the resulting semi-structured data. These valuable data, however, are ‘held’ within a single review.

Further and extraneous use of these semi-structured study data is not easy as exporting them from reviews once published is problematic. Exports are partial and limited to a format specific to RevMan. Full replication of the review analyses is impossible and the use for novel purposes very limited. Wider access to the semi-structured data affords opportunity for auto-curation—automatic tidying of the data—to either return to the source file in a clearer form, or for import into other reviews or programmes for reuse or novel analyses. Cochrane can supply full datasets on request but access to these has been limited. Recent encouragement for the Cochrane organisation to honour their own commitments to wide access to the basic dataset is welcome [3], but the practical difficulty remains of how to easily acquire data in an accessible form.

## Aim

To describe an open-access software programme that facilitates supplying, structuring and curation of these data.

### Procedure

#### Resource

One interested undergraduate student (LS) of Applied Health Sciences, with past experience of RevMan files [4] (4 months full time), access to 224 pre-publication RevMan files and the help of printed popular texts [5], numerous YouTube tutorials and internet fora [6]. For creating the RAPTOR tool, the integrated development environment ‘Eclipse’ was used (Oxygen.3a version) [7].

#### Programming

**Fig. 1:**
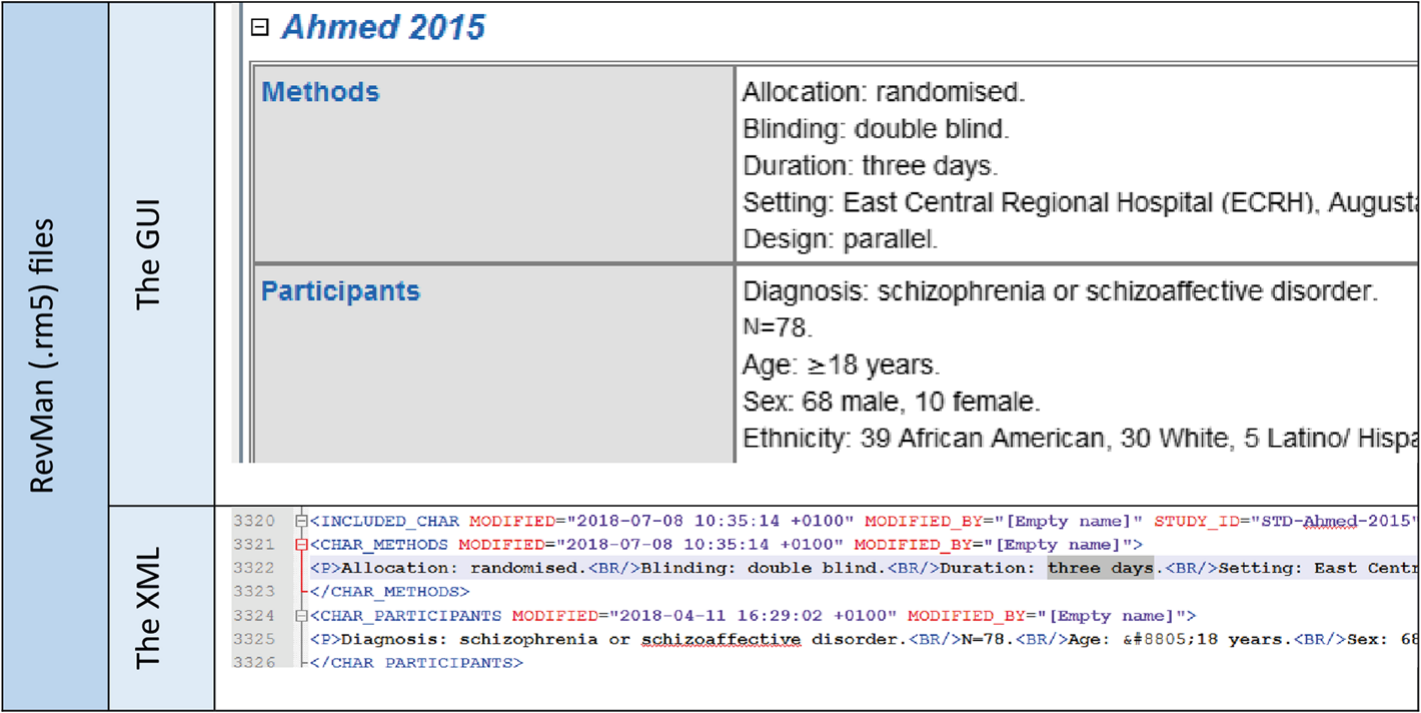
Relation between study data and XML

RevMan (v5.3), the open-access text editor for authors of reviews [2], produces structured XML (Extensible Markup Language) files (.rm5) within which there are structured references to reports of studies (often a many-to-one relationship), one semi-structured table per study containing details of the methods, participants, interventions and outcomes of that study (Fig. 1); one other semi-structured risk of bias table per study; and, finally, sets of tables containing the labelled numerical outcome data of that study (such as outcome name, number of events). PRISMA study flow diagrams, as well as final meta-analysis results and figures, are ignored as we were unsure if these RevMan-processed data were public domain. The internal structure of .rm5 files is regulated. This makes it possible to read content automatically and to process many files in one batch.

For each variable, a path for extraction from the file‘s XML ‘backend’ (Fig. 1) was identified and RAPTOR parses, extracts, sometimes analyses and marshals all variables back to a newly created, central XML file. When analysing prose content, mostly from the ‘Characteristics of included studies’ tables, the text is parsed or split into relevant variables and analysed using regular expressions [8] (regex). These regex, built into the code of RAPTOR, are based on a preliminary content analysis of schizophrenia reviews. With basic knowledge of Java, they can be adjusted to perform more in-depth analyses or replaced by regex tailored to other review group’s conventions and terminology.

In this way RAPTOR has the capacity to recognise the very many ways of reporting the same variable and automatically tidy the final data set. For example, whether a study is double blind can be expressed in many ways (‘double blind’, ‘double-blind’, ‘doubly blinded’ etc.), and RAPTOR extracts these as ‘double’ to the XML Element ‘Blinding’. ‘Double’ could equally well have been given a numeric code.

The free text titles of *outcomes* within RevMan were a problem. In 300 Cochrane schizophrenia reviews, there were over 13,000 unique titles (despite much effort to encourage consistency of reporting) extracted by RAPTOR directly from the reviews without the need of regex or other adjustments. The 13,000 were then manually parsed (within MS Excel) by an experienced reviewer (CEA) to result in a proper ontology of usable (and re-usable) outcomes (15 categories, with variations and sub-categories). Again RAPTOR has the capacity to store the thousands of ways of labelling outcomes and then cluster and output these in a way that led to the creation of an acceptable ontology. For example, see Fig. 1.

**Fig. 2:**
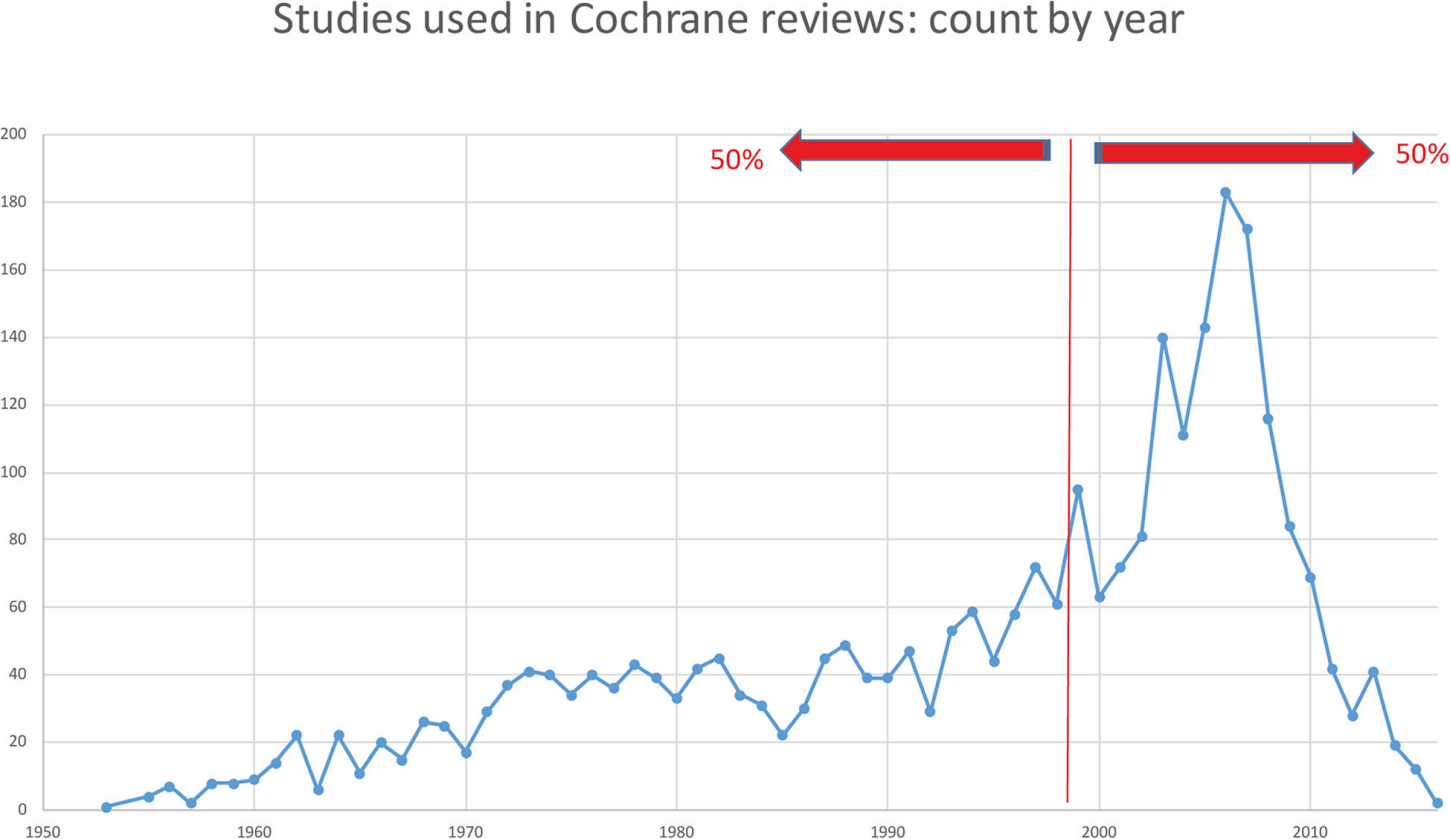
Publication years of studies included in Cochrane schizophrenia reviews

The qualitative and quantitative data, extracted from each study record within the .rm5 file, now more uniform, are again in XML format and can easily be read and queried by many commonly used packages other than RevMan (Fig. 2).

## Results

The RAPTOR programme now exists as a proof of concept. It illustrates how all data can be extracted from large batches of ReviewManager files, and, partly, how these data can be cleaned. Additionally, it has been possible to feed curated qualitative data back into the source review to improve clarity and formatting. This is shown here in an adapted version of RAPTOR that collects the names of all outcomes to which a study contributed. It then appends this list to the relevant section of the study’s characteristics table, a task that is normally undertaken by hand and a common source of errors and inconsistencies [9].

RAPTOR’s output is a XML file which can be queried, for example, in Excel or uploaded to be shared on, for example Google Cloud [10]. One big dataset file generated from all reviews can be uploaded unto Google Cloud Platform and swiftly queried for novel purposes with BigQuery [11] and explored in the Data Studio [12]. A simple example is given below where it took seconds to disclose that half the studies in Cochrane Schizophrenia reviews are over two decades old.

## Discussion

No matter how well-trained reviewers are or how careful editing and copy-editing is, free text boxes lend themselves to inconsistency of reporting. This can only be partly accounted for a program such as RAPTOR. More resource would help but this would remain an issue. Nevertheless, RAPTOR does extract all relevant data into a format that can be queried. This open-access programme batch processes hundreds of reviews within seconds. Whilst the free text is preserved, RAPTOR also automatically edits to produce a more consistent, tidy, text-based output.

There are many limits to RAPTOR’s functionality, but, nevertheless, it opens up so many possibilities for improvement in the logistics and science of reviewing. The XML output affords opportunity for considerable possibilities; some of which are listed as examples and in Table 1:

- This format can link directly, through unique ID, with other databases such as:
  - Registers of studies—and then on through to full text of reports, opening the path between extracted data and source. If necessary, each piece of qualitative and quantitative data could then be hyperlinked to and thus verified from its original report source.
  - Registers of outcome scales—in this way, outcomes employing these scales could have well-written, referenced explanations of what they measure made accessible to readers of the review.
- The well-structured data, even as they currently output from RAPTOR, could be used to draft sections of existing reviews, as implemented in this example [9]. For example, in RevMan’s current ‘Results’ section, when describing the studies within the review, authors are often left to manually total data (e.g. the number of participants)—an unnecessary, tedious and mistake-prone task. Averaging could be easy for many variables once they are structured.
- The return journey from RAPTOR XML into RevMan files can also result in the automatic tidying of the semi-structured data and consequent improvement of reviews.
- The journey of these extracted data out from other works is also possible. Verification of review findings through replication is much simpler (and cost-efficient) with many ways for new use of these valuable data opening up.

**Table 1:** Range of users and use cases

User	Suggested purposes
Editors	Gaining insights on bigger scale trial demographics in a discipline, such as frequent outcomes, years when included studies were published, etc.
Reviewers	Saving time with extracting and assessing trials because data can be reused.
Information specialists	Saving time with extracting and assessing trials because data can be reused.
Other researchers	Any researcher could use the xml file or any data spreadsheet that is made available. This includes people interested in machine learning, classifiers and neural networks approaches who need big amounts of labelled training data. PICO classifiers can be optimised using the characteristics and outcome data. Bias assessment data can be used for similar purposes.

## Conclusions

In most cases, the RAPTOR XML data is not the ‘Big data’ of all individual patient data (IPD) from within trials. Rather, they are the tidied, reliably and verifiably sourced ‘composite’ trial data. Programmes such as RAPTOR make these data widely accessible with the potential for greatly reducing drudgery, duplication and costs of reviewing, whilst increasing quality, efficiency and innovation.

## Data Availability

RAPTOR is available to download (https://github.com/CochraneSchizophrenia/RAPTOR)—including the source code. For feeding data back into reviews, source code is available under (https://github.com/CochraneSchizophrenia/OutcomesApp).

## Abbreviations

NIHR: National Institute of Health Research
RAPTOR: RevMan Parsing Tool for Reviewers
RevMan: ReviewManager (5.3)
XML: Extensible Markup Language
